# Synthesis and Fluorescence Properties of New Ester Derivatives of Isothiazolo [4,5-b] Pyridine

**DOI:** 10.1007/s10895-015-1504-6

**Published:** 2015-01-23

**Authors:** Edward Krzyżak, Małgorzata Śliwińska, Wiesław Malinka

**Affiliations:** 1 Department of Inorganic Chemistry, Wrocław Medical University, ul. Borowska 211A, 50-556 Wrocław, Poland; 2 Department of Chemistry of Drugs, Wroclaw Medical University, ul. Borowska 211, 50-556 Wrocław, Poland

**Keywords:** Synthesis, Fluorescence, DFT, Excited state

## Abstract

A two new compounds with potential biologically active were synthesized: ethyl 4-(2H-4,6-dimethyl-3-oxo-2,3-dihydroisothiazolo [5,4-b] pyridin-2-yl) butanoate and ethyl 4-(2H-4,6-dimethyl-2,3-dihydroisothiazolo [5,4-b] pyridin-3-yloxy) butanoate. The structures of all of the newly formed compounds were identified by elemental analysis, FTIR and ^1^H NMR. Their optical properties were studied in ethanol and n-hexane by UV–Vis absorption and fluorescence spectroscopy. The ground-state and excited-state properties were investigated using the density functional theory (DFT) and the time-dependent density functional theory (TDDFT) methods. The results showed differences between emission spectra in ethanol and n-hexane solution (solvatochromism) for both new compounds.

## Introduction

The interest in the fluorescent molecules has steadily increasing in recent years. Fluorescent biomarkers and probes provide in-depth knowledge about biological system. Many scientific articles have focused on the absorption and emission properties of differently substituted benzoxazoles and benzothiazoles because of their high quantum yields. They have a wide range of applications as fluorescent probes and as intermediates for dyes [1–4]. Although a number of papers have been published concerning the fluorescent properties of different heterocyclic systems, isothiazolo [4,5-*b*] pyridine moiety has not yet been extensively studied.

Here we report the synthesis and fluorescence properties of novel derivatives of isotiazolo [5,4-*b*] pyridine. Early studies demonstrated that some of isotiazolopyridines containing different substituents have been investigated because of their biological activity. Their activity depends usually on nature of substituents attached to the isothiazole ring. For example, 2-(4-substituted-piperazin-1-ylalkyl)-3-oxoisothiazolo [5,4-*b*] pyridines display strong analgesic activity [5, 6] and moderate antimycobacterial properties [7]. Other authors have also suggested antiagregative action of derivatives of isothiazolo [5,4-*b*] pyridine [8].

On the other hand, the literature reports examples of antinociceptive agents bearing an alkanoic acid or ester moieties linked to the 3-nitrogen of 2-oxo-3*H*-benzoxazole ring [9, 10]. Gulcan and co-workers [10] synthesized 4-(5-chloro-2(3*H*)-benzoxazolon-3-yl) butanoic acid and its ethyl ester with analgesic activity that was higher or comparable to that of aspirin and, what is more, without gastric side effect.

Taking into account the interesting biological activity of isothiazolopyridines and related benzoxazoles, we have described novel ethyl 4-(2*H*-4,6-dimethyl-3-oxo-2,3-dihydroisothiazolo [5,4-*b*] pyridin-2-yl) butanoate and its 3-O-substituted isomer. The aim of this work is to synthesize and investigate the basic fluorescence properties of new compounds. The presence of strong basic and electron deficient pyridine ring in the isothiazolo [4,5-*b*] pyridine moiety makes these systems different from the better known benzoxazole and benzothiazole rings.

## Experimental

### Materials

All reagents and solvents were purchased from commercial suppliers. Dry solvent was obtained according to the standard procedure. Flash column chromatographic purifications were performed using Sigma-Aldrich 60A silica gel 230–400 mesh. Progress of the reaction was monitored by TLC on silica gel 60 F254-coated TLC plates (Fluka Chemie Gmbh) and visualized by UV light at 254 nm.

### Methods and Instruments

The proton nuclear magnetic resonance (^1^H NMR) spectra were recorded on a Brucker 300 Mhz NMR spectrometer using tetramethylsilane (TMS) as internal reference. Chemical shifts are reported as *δ* in parts per million (ppm). The samples were prepared by dissolving 5 mg of each form in 600 μl of CDCl_3_.

FTIR spectra were run on a Perkin-Elmer Spectrum Two UATR FT-IR spectrometer. The samples were applied as solids.

Thermal characteristic was carried out on a Mettler Toledo DCS 25 measuring cell with TC15 TA Controller, calibrated with indium to ensure the accuracy of the calorimetric scale. Samples weighing 3 mg were characterized in sealed 40 μL aluminium pans and subjected to thermal analysis under a flowing argon atmosphere (30 cm^3^ min^−1^), using heating rate of 5 °C min^−1^.

Elemental analyses for carbon, nitrogen and hydrogen were carried out on an Carlo Erba NA 1500 analyser and were within ± 0.4 % of the theoretical value.

### Computational Methods

The electronic structure calculations were carried out using Gaussian 09 program package [11]. The ground state geometric optimizations was calculated using density functional theory (DFT) with Becke’s three-parameter hybrid exchange function with the Lee–Yang–Parr gradient corrected correlation (B3LYP) [12–14] functional in combination with 6–311 + G (d, p) basis set. The electronic properties, such as absorption and emission wavelengths, oscillator strengths, were calculated using time-dependent density functional theory at the TDDFT/6–311 + G (d, p) level. The hybrid functionals PBE0 [15] were employed in calculations.

## Results and Discussion

### Synthesis

**Fig. 1:**
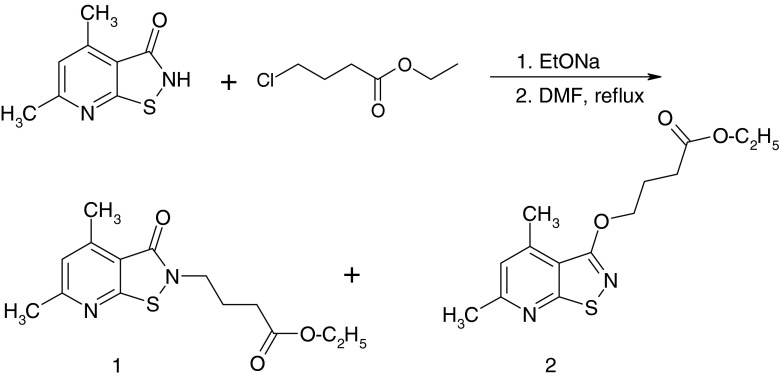
Scheme for the preparation of Ethyl 4-(2H-4,6-dimethyl-3-oxo-2,3-dihydroisothiazolo [5,4-b] pyridin-2-yl) butanoate **(1)** and Ethyl 4-(2H-4,6-dimethyl-2,3-dihydroisothiazolo [5,4-b] pyridin-3-yloxy) butanoate **(2)**

Procedure for the preparation of *Ethyl 4-(2H-4,6-dimethyl-3-oxo-2,3-dihydroisothiazolo [5,4-b] pyridin-2-yl) butanoate (1) and Ethyl 4-(2H-4,6-dimethyl-2,3-dihydroisothiazolo [5,4-b] pyridin-3-yloxy) butanoate (2)* (Fig. 1).

1,08 g (6 mmol) of 4,6-dimethyl-3-oxo-2,3-dihydroisothiazolo [5,4-*b*] pyridine [16] was dissolved in an ethanolic solution of sodium ethoxide prepared by the reaction of Na° (0,14 g, 6 mmol) with 30 mL of anh. ethanol. After evaporation to dryness under vacuum, the residue was dissolved in 30 mL N, N-dimethylformamide and then 0,99 g (6,6 mmol) of ethyl 4-chlorobutanoate was added, heated for 12 h., cooled to room temperature and poured into 50 mL ice-water. The precipitate was collected by suction filtration. The residue was chromatograhed [CC; ethyl acetate:cyclohexene:chloroform (2:2:1)]. The fractions containing the product of R_f_ = 0,83 afforded 1,06 g of **2** (yield: 60 %), whereas fractions of R_f_ = 0,55 gave additionally 0,53 g **1** (yield: 30 %).

The final compounds were characterizes by FTIR and ^1^H NMR methods. Selected results are given below.

**1:** *Anal.* C_13_H_16_N_2_O_2_S (m.w. 294,39); ^1^H NMR (CDCl_3_) *δ*: 1,25 t (3H, OCH_2_C**H**_3,_ J = 7,2 Hz), 2,03-2,13 m(2H, CH_2_C**H**_2_CH_2_), 2,40 t(2H, CH_2_C**H**_2_CO, J = 7,5 Hz), 2,60 s(3H, CH_3_), 2,74 s(3H, CH_3_), 3,92 t(2H, NCH_2_, J = 7,2), 4,12 q(2H, OC**H**_2_CH_3_, J = 7,2), 6,94 s(1H, ArH). FT-IR (UATR, selected lines): 1720 (C = O, ester), 1670 (3-C = O) cm^-1^. Anal. Calcd: C, 57,11; H, 6,17; N, 9,52. Found: C, 57,10; H, 6,32; N, 9,35.

**2:** *Anal.* C_13_H_16_N_2_O_2_S (m.w. 294,39); ^1^H NMR (CDCl_3_) *δ*: 1,25 t (3H, OCH_2_C**H**_3,_ J = 7,2 Hz), 2,18-2,24 m(2H, CH_2_C**H**_2_CH_2_), 2,52 t(2H, CH_2_C**H**_2_CO, J = 7,5 Hz), 2,63 s(3H, CH_3_), 2,66 s(3H, CH_3_), 4,13 q(2H, OC**H**_2_CH_3_, J = 7,2), 4,54 t(2H, OC**H**_2_CH_2_, J = 6), 6,93 s(1H, ArH). FT-IR (UATR, selected lines): 1720 (C = O, ester) cm^-1^. Anal. Calcd: C, 57,11; H, 6,17; N, 9,52. Found: C, 57,46; H, 6,15; N, 9,52.

**Fig. 2:**
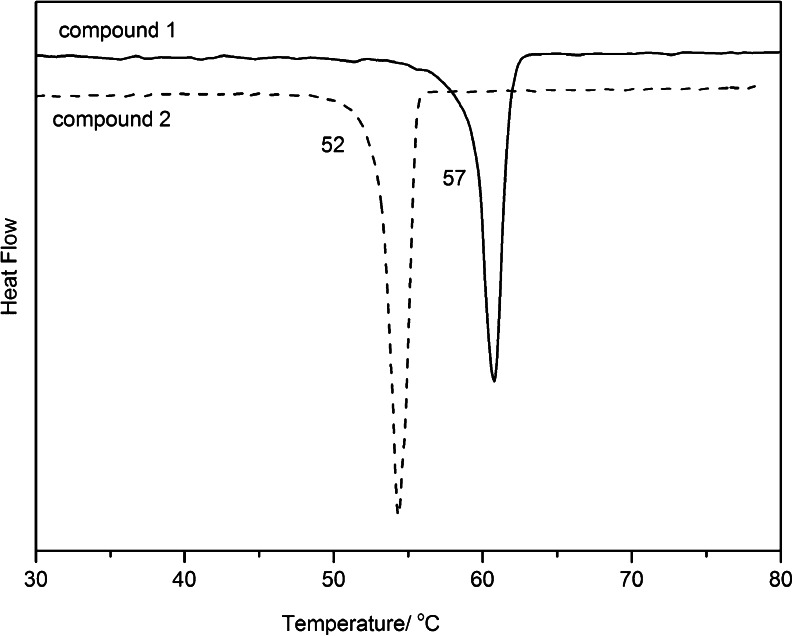
DSC curves for studied compounds heated at 5 °C min^−1^

Additionally, the compounds were analyzed by differential scanning calorimetry. The DSC trace shows only one thermal effect (Fig. 2). It corresponds to melting process. The melting temperature was determined as 57 °C for compound 1 and 52 °C for compound 2.

### UV–vis Absorption Spectra

**Fig. 3:**
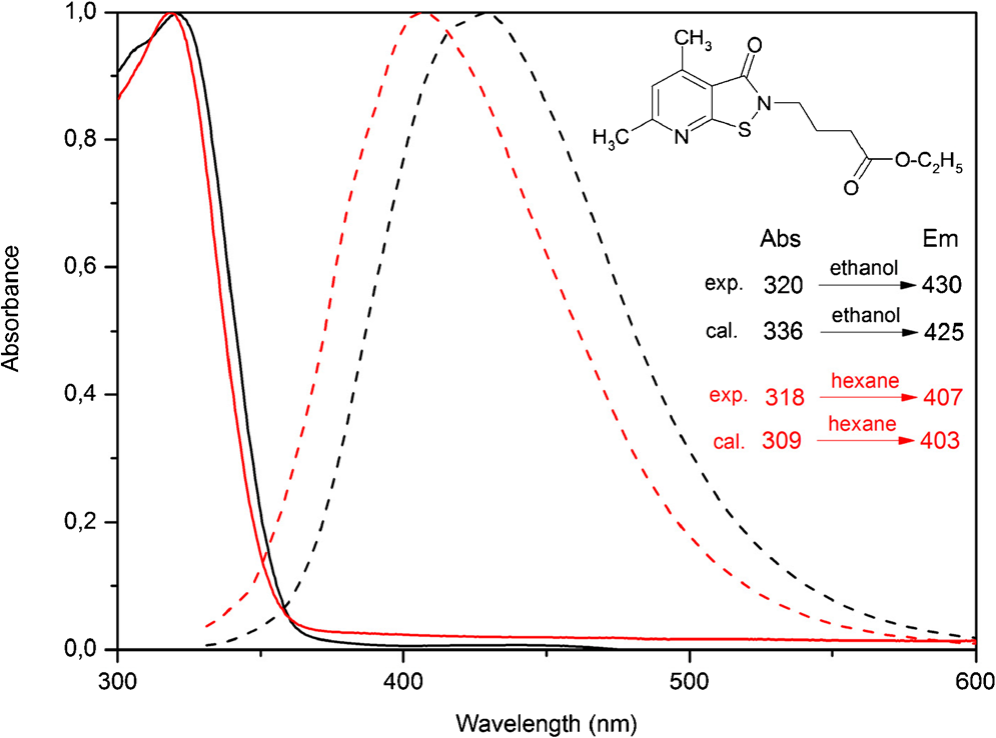
Normalized UV–Vis (*solid line*) and emission (*dot line*) spectra in ethanol (*black*) and n-hexane (*red*) solution for compound 1

**Fig. 4:**
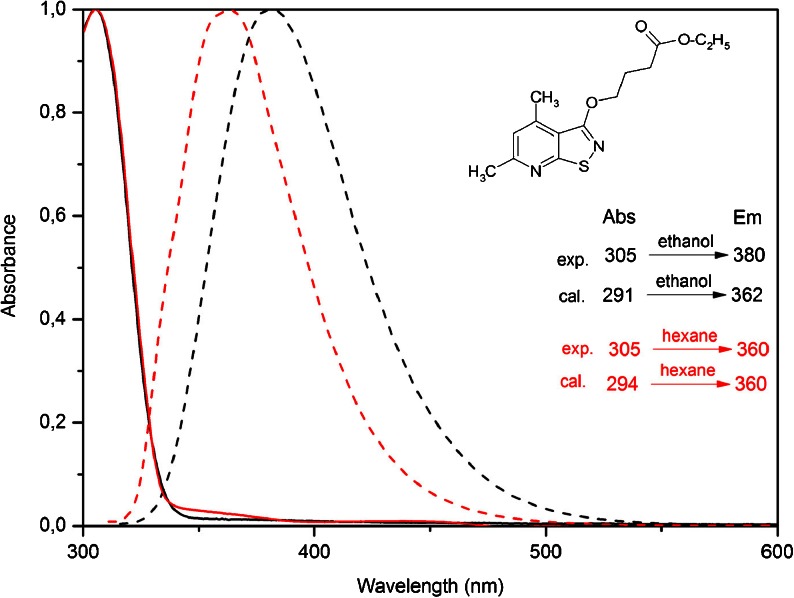
Normalized UV–Vis (*solid line*) and emission (*dot line*) spectra in ethanol (*black*) and n-hexane (*red*) solution for compound 2

The experimental UV–Vis spectra in ethanol and n-hexane for compound 1, 2 are shown in Figs. 3 and 4 and their spectra data are summarized in Table 1. The results showed strong broad absorption bands starting at 365 nm with maximum at 320 (ethanol) and 318 nm (n-hexane) for compound 1 and 345 nm with maximum 305 nm for compound 2. The calculated absorption maxima values have been found to be 336 (ethanol), 309 nm (n-hexane) for compound 1 and 291 (ethanol), 294 nm (n-hexane) for compound 2.

**Table 1:** Experimental and calculated spectra in ethanol and n-hexane solution for Ethyl 4-(2H-4,6-dimethyl-3-oxo-2,3-dihydroisothiazolo [5,4-b] pyridin-2-yl) butanoate **(1)** and Ethyl 4-(2H-4,6-dimethyl-2,3-dihydroisothiazolo [5,4-b] pyridin-3-yloxy) butanoate **(2)**

Compound	UV–Vis spectra λ_max_(nm)		Fluorescence spectra λ_max_(nm)	
	Ethanol	n-hexane	Ethanol	n-hexane
1	exp. 320 cal. 336	exp. 318 cal. 309	exp. 430 cal. 425	exp. 407 cal. 403
2	exp. 305 cal. 291	exp. 305 cal. 294	exp. 380 cal. 362	exp. 360 cal. 360

### Fluorescence Spectra

**Fig. 5:**
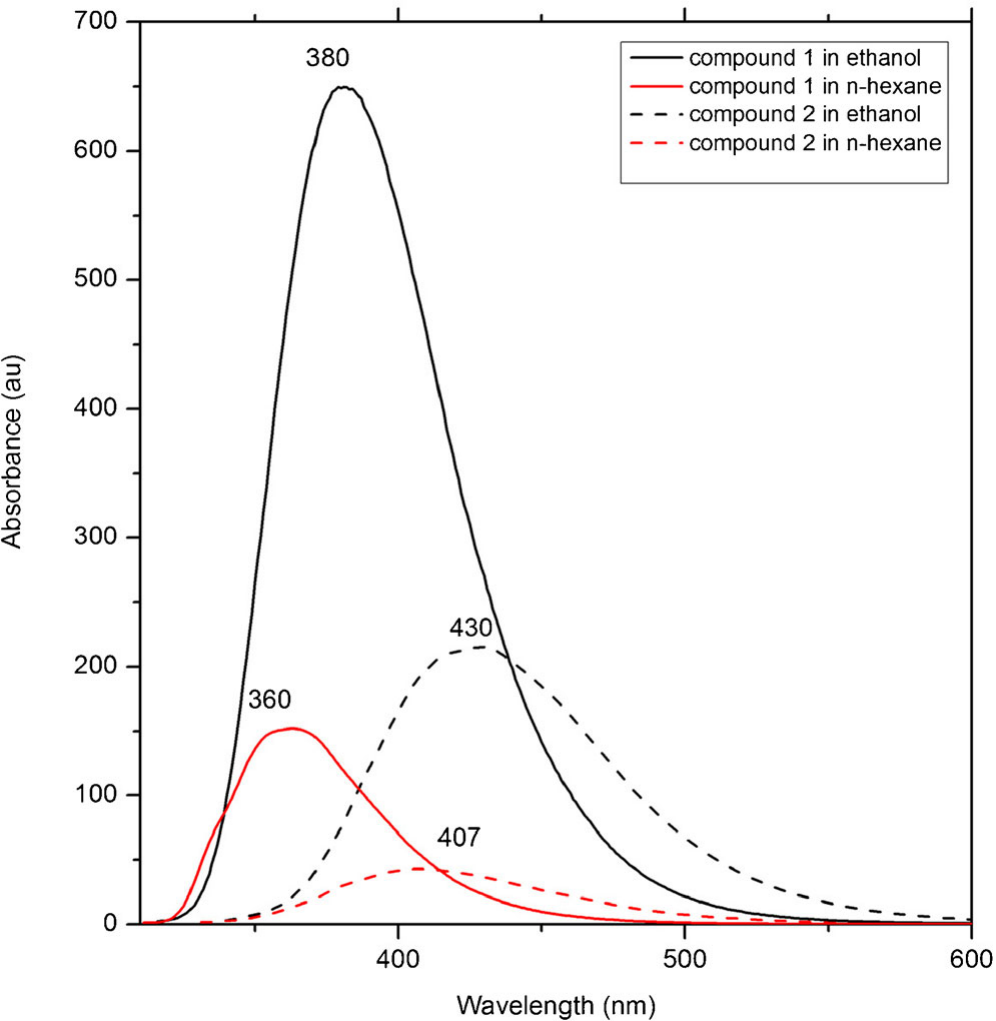
Experimental emission spectra in ethanol (*black*) and n-hexane (*red*) solution for compound 1 (*solid line*) and compound 2 (*dot line*)

The fluorescence spectra was recorded in ethanol and n-hexane at a concentration of 1.0 × 10^-5^ mol dm^-1^. Their emission spectra are shown in Figs. 3 and 4 and their spectra data are summarized in Table 1. The maximal emission peaks of compound 1 are located at 407 nm in n-hexane solution and 430 nm in ethanol solution. The fluorescence intensity in ethanol solution is much higher (Fig. 5). The calculated emission maxima values have been found to be 425 (ethanol), 403 nm (n-hexane) and they are in good accordance with experimental. For compound 2, the maximal emission peaks are located at 360 nm (n-hexane) and 380 nm (ethanol). The calculated values are 360 and 362 nm, respectively. The fluorescence intensity in ethanol solution is much higher, similarly as for compound 1 (Fig. 5).

Fluorescence quantum yields were determined in different solvents by using anthracene (Φ = 0.36 in cyclohexane) as a reference standard using the comparative method. The fluorescence quantum yields of the synthesized compounds were 0.15 (ethanol) and 0.10 (n-hexane) for 1 and 0.10 (ethanol) and 0.07 (n-hexane) for 2.

### Calculations

**Fig. 6:**
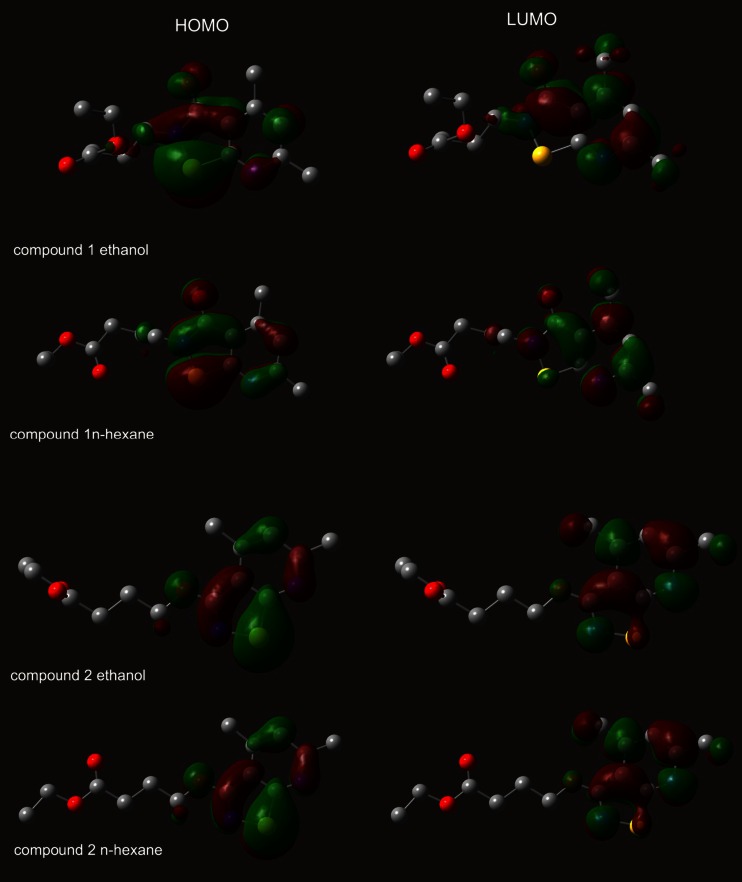
The plots of HOMO and LUMO orbital contour for molecule 1 and 2

Based on our theoretical results the fluorescent state of both compounds corresponds to the orbital transition from the highest occupied molecular orbital (HOMO) to the lowest unoccupied molecular orbital (LUMO). The atomic orbital compositions of the frontier molecular orbital are sketched in Fig. 6. In the HOMO, the charge density is mainly accumulated on the pyridine ring. In case of the LUMO, more charge density moves to the isothiazole part. The transitions which are observed are assigned as π → π*. As shown in Figs. 2 and 3, the value of maximum absorption peaks for the first band is almost identical in both solutions and different for N-isomer and O-isomer. For both isomers, the fluorescence spectra was found to be dependent on solvent polarity and intensity in ethanol solution is much higher than n-hexane (Fig. 5). As is well known with increasing solvent polarity, the ground state molecule is better stabilized by solvation than the molecule in the excited state (blue shift will result), or better stabilization of the molecule in the first excited state relative to the ground state, with increasing solvent polarity, will lead to positive solvatochromism (red shift). An important parameter is the dipole moment difference between the ground and excited state. The dipole moment calculated within TDDFT formalism for the N-isomer (compound 1) in the ground state is found as 2.05 D in the n-hexane solution, and increases to 2.88 D in excite state. In the ethanol solution, the dipole moment is slightly higher and increases from 3.26 D (ground state) to 4.25 D (excite state). For the O-isomer (compound 2) the dipole moments are slightly higher: in the ground state 3.27 D and excite state 4.08 D (n-hexane solution) and 5.80 D, 6.92 D, respectively in ethanol solution. The difference between the ground and excited state is rather small in all cases: ∆μ = 0.83, 0.99, 0.81 and 1.12 D, respectively. Those calculations are in good agreement with experimental absorption spectra. However, the emission spectra in ethanol solution is red shifted. A variety of environmental factors affect fluorescence emission, including interactions between the molecule and surrounding solvent. Solvent molecules assist in stabilizing and further lowering the energy level of the excited state by re-orienting around the excited fluorophore and in the results reducing the energy separation between the ground and excited states. Increasing the solvent polarity (ethanol) reduces the solvent effect on the excited state energy level. Our calculation also does not include the intermolecular interactions. Moreover, both isomers have atoms with free electron pairs (O and N). Therefore the transitions can have mixed character: n → π* and π → π*, and affect the emission spectra and solvent effect.

## Conclusion

In this work, two new ester derivatives of isothiazolo [4, 5-*b*] pyridine, compounds with potential biologically active, were synthesized. Their structures were confirmed by FTIR, ^1^H NMR, elemental analysis techniques. The absorption and emission spectra of titled compounds in ethanol and n-hexane solution were studied experimentally and theoretically. The results showed that the fluorescence spectra depends on solution. The emission spectra in ethanol solution is red shifted. The calculated dipole moment increases in excited state and is higher in ethanol than n-hexane solution. Polarity of the solvent also affects the intensity of the emission band. The results obtained clearly show differences in optical properties of N- and O-isomer. Due to light emission properties and ease methods of synthesis, the new derivatives may be considered for the development of functional dyes.
